# Stimulation of regulatory dendritic cells suppresses cytotoxic T cell function and alleviates DEN-induced liver injury, fibrosis and hepatocellular carcinoma

**DOI:** 10.3389/fimmu.2025.1565486

**Published:** 2025-04-08

**Authors:** Junjie Wang, Pixu Gong, Qingqing Liu, Menglei Wang, Dengfang Wu, Mengyu Li, Shujie Zheng, Han Wang, Qiaoming Long

**Affiliations:** ^1^ Jiangsu Key Laboratory of Neuropsychiatric Diseases and Cam-Su Mouse Genomic Resources Center, Suzhou Medical College, Soochow University, Suzhou, Jiangsu, China; ^2^ Center for Circadian Clocks, Soochow University, Suzhou, Jiangsu, China; ^3^ School of Basic Medical Sciences, Suzhou Medical College, Soochow University, Suzhou, Jiangsu, China

**Keywords:** TLR2, rDCs, T cells, hepatocellular carcinoma, lactic acid producing probiotics

## Abstract

**Background:**

Dendritic cells (DCs) are versatile professional antigen-presenting cells and play an instrumental role in the generation of antigen-specific T-cell responses. Modulation of DC function holds promise as an effective strategy to improve anti-tumor immunotherapy efficacy and enhance self-antigen tolerance in autoimmune diseases.

**Methods:**

Wild-type (WT) and TLR2 knockout (KO) mice at 2 weeks of age were injected intraperitoneally (i.p.) with a single dose of diethylnitrosamine (DEN) to induce hepatocellular carcinoma (HCC). Four weeks later, WT and KO mice were randomly divided into control and treatment groups and treated once every two days for 30 weeks with phosphate buffered saline (PBS) and a mix of 4 TLR2-activating lactic acid-producing probiotics (LAP), respectively. Mice were euthanized after 30 weeks of LAP treatment and their liver tissues were collected for gene expression, histological, flow cytometric and single-cell RNA sequencing analyses.

**Results:**

We demonstrate here that oral administration of a mix of TLR2-activating LAP triggers a marked accumulation of regulatory DCs (rDCs) in the liver of mice. LAP-treated mice are protected from DEN-induced liver injury, fibrosis and HCC in a TLR2-dependent manner. Single-cell transcriptome profiling revealed that LAP treatment determines an immunosuppressive hepatic T-cell program that is characterized by a significantly reduced cytotoxic activity. The observed functional changes of T cells correlated well with the presence of a hepatic DC subset displaying a regulatory or tolerogenic transcriptional signature.

**Conclusion:**

Overall, these data suggest that stimulation of regulatory dendritic cells (rDCs) in the liver by LAP suppresses cytotoxic T-cell function and alleviates DEN-induced liver damage, fibrosis and tumorigenesis.

## 1 Introduction

Hepatocellular carcinoma (HCC) is the most common primary liver malignancy. Worldwide, HCC accounts for over 800,000 deaths annually, the second leading cause of cancer-related mortality (1). HCC usually develops in the setting of chronic hepatitis and cirrhosis, conditions that are causally associated with a viral infection, alcohol consumption, endotoxin as well as metabolic dysfunction-related liver injuries (2, 3). These conditions result in hepatocyte death and compensatory hepatocyte proliferation, which, together with endoplasmic reticulum and oxidative stress, drive hepatocarcinogenesis (4–6). The global prevalence of HCC is rapidly increasing, a direct effect of the growing worldwide obesity epidemic (7). Traditional treatment options for HCC include surgical removal, local ablation, chemo- and radiotherapy (3). Therapies targeting the programmed death 1 (PD-1) and cytotoxic T lymphocyte-associated antigen 4 (CTLA-4), the immune checkpoints, have shown unprecedented rates of durable clinical responses in patients with several solid and hematological cancers (8, 9). Despite this, only a subset of HCC patients shows favorable responses to PD-1 and CTLA-4-based immunotherapies (10), underscoring the need for a deeper understanding of the cellular and molecular mechanisms underlying HCC pathogenesis, in particular the roles of hepatic immune cells.

The liver is populated by a variety of immune cells, including macrophages (Kupffer cells, KC), dendritic cells (DC), natural killer (NK) cells, neutrophils, B and T lymphocytes (11). These distinct innate and adaptive immune cells form a sophisticated immune surveillance network to protect hepatocytes against invading pathogens and from chemically or metabolically triggered hepatocellular damages (12). Growing evidence from liver disease patients and murine models indicated that dysfunction and/or dysregulation of the hepatic immune cell system plays an essential role in the pathogenesis of liver fibrosis and cirrhosis, and consequently, HCC, by producing proinflammatory cytokines such as TNFα, IL-1β and IL-6, to drive necroinflammation and hepatocyte death (4–6). From a therapeutic perspective, targeted manipulation of specific immune cells subsets, such as tumor-associated macrophages (TAMs) and neutrophils (TANs), may offer effective strategies to prevent hepatic inflammation and cell death, thus novel treatments for liver cancer (13–15).

Dendritic cells (DCs) are a diverse group of specialized immune cells developed from bone marrow hematopoietic precursors (16). DCs have been well-recognized for their ability to present various self and non-self-antigens in conjunction with major histocompatibility complex (MHC) molecules to naïve T lymphocytes to prime T cell responses (17, 18), qualifying them as essential mediators of systemic or tissue-specific adaptive immune responses. As such, there has been a persistent interest over the past few decades in developing DC-based treatment strategies for various cancer types, including HCC (18, 19), especially following the remarkable patient responses observed with novel checkpoint blockade therapies (20). It is noteworthy that aging decreases the migrating and cytokine-producing abilities of DCs, thereby negatively impacting the anti-tumor and anti-viral adaptive immune responses in elderly mice and humans (21). Of note, correcting DCs migration defect using a vaccine adjuvant reverses aging-related adaptive immune defects and improves anti-tumor immunity in aged mice (22). Thus, modulating the cross-presenting function of the DC subset represents a promising tool for improving the efficacy of next-generation cancer immunotherapies.

rDCs, are commonly found in the microenvironment of advanced solid tumors (23, 24). This discovery has fundamentally shifted the perception of DCs solely as inducers of immune reactivity. As such, DCs are now recognized to have the potential to both stimulate and inhibit immune responses (25, 26). Tumor-associated rDCs may directly or indirectly maintain antigen-specific or non-specific T cell unresponsiveness by controlling T cell polarization, myeloid-derived suppressive cell (MDSC) and regulatory T cell (Treg) differentiation and activity, consequently leading to tumor initiation and progression (23, 27). Despite these understandings, the molecular nature and function of rDCs, as well as their relationships with other myeloid and T cell subsets during HCC development, remain largely unknown thus far.

Probiotics are popular food supplements and have shown potent immunostimulatory effects in both healthy subjects (28) and gastrointestinal cancer patients (29). certain probiotic strains have also demonstrated beneficial roles of in lowering systemic inflammation and in suppressing extraintestinal tumor growth, doing so at least in part through either inhibiting T helper 17 (Th17) cell differentiation or stimulating rDC formation (30, 31). The present study aims to determine whether and how hepatic DC manipulation affects diethylnitrosamine (DEN)-induced HCC formation in mice. We show that daily oral administration of LAP, a novel mix of four live lactic acid-producing probiotics, mitigates DEN-induced liver injury, reduces hepatic fibrosis and suppresses HCC progression. The hepatoprotective effect of LAP is associated with an expanded DC population in the liver. Single-cell RNA profiling reveals that LAP treatment causes a markedly repressed cytotoxic T-cell program in the liver. Gene expression analysis indicates that the expanded hepatic DC subsets broadly display a transcriptional signature indicative of regulatory dendritic cells. Overall, our findings suggest that targeted stimulation of rDCs in the liver protects against DEN-induced tumorigenesis by attenuating T cell-mediated hepatocyte death.

## 2 Materials and methods

### 2.1 Animal experiments

In this project, C57BL/6 WT mice were purchased from Gempharmatech Co., Ltd (Nanjing, China), and TLR2 KO mice were a gift from the lab of S. Xiong (Soochow University) and bred on C57BL/6 mice.

2-week-old male mice were injected (i.p.) with a single dose of 25 mg/kg diethylnitrosamine (DEN; Sigma N0258), then fed with high-fat diet and provided with probiotics by gavage (i.g.) at 6 weeks of age, finally euthanized and harvested with tumor for analysis at 36 weeks of age. Tumor volume = length x width^2 x 1/2.

All mice were housed in a Specific Pathogen Free (SPF) facility and all animal operations were performed in accordance with the protocol approved by the Animal Ethics Committee of Soochow University.

### 2.2 TLR2 reporter-based probiotics screening

Lactobacillus plantarum WCFS1 (ATCC BAA-793) and Lactobacillus plantarum (BNCC 194165) were purchased from Bena culture collection (BNCC, China). Lactococcus lactis and Lactobacillus plantarum 35 were isolated from a freeze-dried probiotic powder mixture. All probiotics were grown in an MRS medium. For probiotics functional screening, HEK-Dual™ hTLR2 (NF/IL8) cells (InvivoGen) were grown in DEME High Sugar Medium containing 100 ml of DEME High Sugar Medium consisting of 10% FBS, 100 U/mL penicillin G sodium salt, 10 mg/mL streptomycin sulfate. After the cells were inoculated in 96-well plates, 10^7^ CFU of PBS-resuspended bacteria were added and co-cultured for 24 h at 37°C, 5% CO_2_. After 24 h, 10 µL supernatant from each well was incubated with 50 µL Quanti-Luc™ solution, and a microplate reader tested the luciferase value.

### 2.3 Flow cytometry

Minced liver tissues were digested by collagenase 4 for 30 min. The product was filtered through 70 µm cell sieves. Liver parenchymal cells were removed by centrifugation before erythrocytes were removed by LCK lysate. FC blocking was performed at a rate of 1 µL FC block per 1,000,000 cells. After the cells were stained by CD45, CD3, CD8, CD19, Gr-1, CD11b, CD11c, F4/80, NK1.1 Antibody and LIVE/DEAD Fixable Dead Cell Stain Kits, the cells were detected using flow cytometry.

### 2.4 Immunohistochemistry and multiplex immunofluorescence

Dewaxed and hydrated liver tissue sections were antigen retrieved and endogenous peroxidase activity blocked as previously described (32). The sections were then treated with primary antibodies (4°C, 16h) and secondary antibodies (RT, 2h), followed by DAB and hematoxylin staining). Images were acquired using a Nikon digital camera and analyzed by ImageJ. For the immunofluorescence assay, rehydrated liver sections were blocked in 10% goat serum for 2 hours, then incubated with primary antibodies (4°C, 16h) and Polymer-HRP secondary antibody (RT, 30min). After TSA fluorescent dye and DAPI staining, fluorescent images were acquired using a Digital Pathology Scanner (KFBIO, China).

### 2.5 Masson staining

Masson staining was performed according to the manufacturer’s instructions (Solarbio, G1340, China). Briefly, dewaxed and rehydrated liver tissue sections were treated with a weak acid working solution for 30s. The treated sections were then incubated in Phosphomolybic Acid Solution (2min), followed by treatment with Aniline Blue Solution for 2 min. Images were acquired using a Nikon digital camera and analyzed by ImageJ.

### 2.6 qPCR and western blotting

Liver tissue or tumor RNA was extracted using RNAiso Plus (Takara, Japan) and reverse-transcribed using a HiScript III 1st Strand cDNA Synthesis Kit (Vazyme, China). Quantitative PCR was performed using SYBR Green (Vazyme, China) on a ViiA7 Real-Time PCR system (Applied Biosystems, USA), and β-Actin was used as an internal control. For Western blotting, liver tumor or tissue lysates were prepared as previously described (32). Lysate protein concentrations were determined by BCA assay. Twenty mg of each lysate was resolved in a 10% sodium dodecyl sulfate (SDS)-polyacrylamide gel and then transferred onto a PVDF membrane. The protein-loaded membranes were blocked in 5% milk for 2-4 hours and then incubated with primary and secondary antibodies. Immunodetection was performed using the ECL chemiluminescence kit according to the manufacturer’s specifications. The following antibodies were used: β-tubulin (1:10000) (Proteintech, USA), Bcl-2 (1:1000) (Proteintech, USA), PCNA (1:5000) (Proteintech, USA), Cyclin D1 (1:5000) (Proteintech, USA), CDK4 (1:1000) (Proteintech, USA), ACSL4 (1:2000) (Proteintech, USA), GPX4 (1:1000) (Proteintech, USA), TLR4 (1:4000) (Proteintech, USA), TLR5 (1:1000) (Proteintech, USA) and P-P38 (1:1000) (Proteintech, USA), P-MLKL(1:1000) (CST, USA), MLKL(1:1000) (CST, USA), P-RIP(1:1000) (CST, USA), RIP(1:1000) (CST, USA), TLR2(1:1000) (CST, USA), P38(1:1000) (CST, USA), P-P65(1:1000) (CST, USA), P65(1:1000) (CST, USA) and Caspase3(1:1000) (CST, USA), and TLR9 (1:1000) (Abcam, USA).

### 2.7 Single-cell transcriptome profiling

#### 2.7.1 Library construction and sequencing

Hepatic CD45^+^ cells were prepared through fluorescence-activated cell sorting (FACS). A total of 10000 CD45^+^ cells from 4 mice (2500 cells/mouse) were loaded to a 10 x GemCode Single-cell instrument to generate single-cell Gel Bead-In-Emulsions (GEMs). The GEMs were then subjected to library construction using the Chromium™ Single Cell 3’Reagent Kit (version 3.1) (10X Genomics, Pleasanton, CA) according to the manufacturer’s instructions. Library construction and RNA sequencing were completed by Gene Denovo Biotechnology Co., Ltd. (Guangzhou, China) as described (33, 34).

#### 2.7.2 Data quality control and normalization

Barcode processing, data quality control and normalization were performed using the Cell Ranger Single Cell Software v3.1 (10X Genomics, Pleasanton, CA). Briefly, raw data from the sequencer were demultiplexed into the FASTQ format with the bcl2fastq software and then aligned in the NucleotideSequence Database https://www.ncbi.nlm.nih.gov/genbank/ using the NCBI Basic Local Alignment Search Tool (BLAST). Low-quality sequences (containing adaptor sequences, or “N” longer than 10% of the read) and low-quality cells (containing ≥ 8000 UMIs,≥ 10% mitochondrial genes, and with <500 or >4000 genes detected) were filtered out. After quality control, a dataset of 18,690 CD45^+^ cells (8392 control and 10298 LAP) x 42145 genes was obtained for downstream analysis. The raw gene expression measurements for each cell were normalized by dividing them with the total expression followed by scale factor-multiplying (x10,000) and log-transformation.

#### 2.7.3 Cell clustering and visualization

Data clustering was performed using the Seurat R package v4.0.4. Briefly, filtered and normalized control and LAP datasets were integrated after canonical correlation analysis-based reduction of batch effects. The integrated data were further normalized by the Z-score and then subjected to principle component analysis (PCA) to reduce dimensionality. Subsequently, the enriched PCs with low p-value genes were used in a share-nearest neighbor (SNN) graph approach to cluster cells. The FindCluster tool employing the Louvain algorithm was used to group cells into different subsets according to their expression levels. Single-cell subgroup classification results were visualized by t-distributed Stochastic Neighbor Embedding (t-SNE) using the Louge Cell Browser software. For each cell cluster, genes showing differential expression and with known functions were identified.

#### 2.7.4 Single-cell pseudo-time analysis

Single-cell trajectory analysis was performed with the Monocle v2.10.1 package (35). Briefly, key differentially expressed genes (DEGs) related to the development and differentiation processes were identified by performing differential gene tests and subsequently used as markers to define cellular progress. Data dimension reduction was performed using DDRTress, and cells were ordered in peseudotime using the order-cells function. The trajectory was visualized in a two-dimensional tree-like structure by running the plot cell trajectory function.

#### 2.7.5 Gene functional enrichment analysis

Differentially expressed genes (DEGs) were subjected to Gene Ontology (GO), Reactome and Gene Set Enrichment Analysis (GSEA) to identify biological functions and interacting pathways. GO and Reactome analyses were performed using the Cluster Profiler R package in RStudio (v 1.2.1335) and ClueGO plugin in Cytoscape software (v3.8.2), respectively (36). Outputs with false discovery rate (FDR)-corrected p-value <0.05 were retained. GSEA analysis was performed using GSEA v4.0.3 and thec6.all.v7.0.symbols.gmt (oncogenic signatures) and c2.cgp.v7.0.symbols.gmt (chemical and genetic perturbations) gene setlibraries as reference gene set collections (37). The statistical cutoff for this analysis wasset at p<0.05.

#### 2.7.6 Identification of gene expression programs by cNMF

Gene expression programs underlying cellular activities in hepatocytes, myeloid and T cells were identified using the consensus non-negative matrix factorization (cNMF) method (https://github.com/dylkot/cNMF) (38). Briefly, normalized cell type-specific expression data from control and LAP mice were integrated and used as input to run non-negative factorization (NMF) analysis to identify clusters of highly similar clusters of components inferred as GEPs. This procedure was repeated multiple rounds for each cell type, and a consensus k-value (number of GEPs) was selected, which provided a reasonable trade-off between error and stability. Non-negative least squares (NNLS) was used to calculate the activity of NMF transcription programs in each cell based on the first 100 weighted genes of the GEP (39). Subsequently, Student’s t-test or Mann-Whitney test statistical analysis was performed to compare GEP activity values between control and LAP cells and p<0.05 was defined as statistically different. The top 30 genes of each GEP that show significant activity difference between control and LAP cells were used in GO, KEGG and Reactome analysis to identify the biological functions associated with the GEP (36, 40). Finally, tSNE plots generated with the ggplot2 package were used to visualize the spatial distribution of the statistically different GEPs in cell subtypes.

### 2.8 Statistical analysis

Differences between compared groups were evaluated by performing Student’s t-test or two-way repeated ANOVA using Graphpad (8.0). Data were presented as mean ± standard error, and p<0.05 was considered as significant.

## 3 Results

### 3.1 Dietary supplementation of LAP protected wild-type but not TLR2 knockout mice from DEN-induced liver injury, fibrosis and tumorigenesis

**Figure 1:**
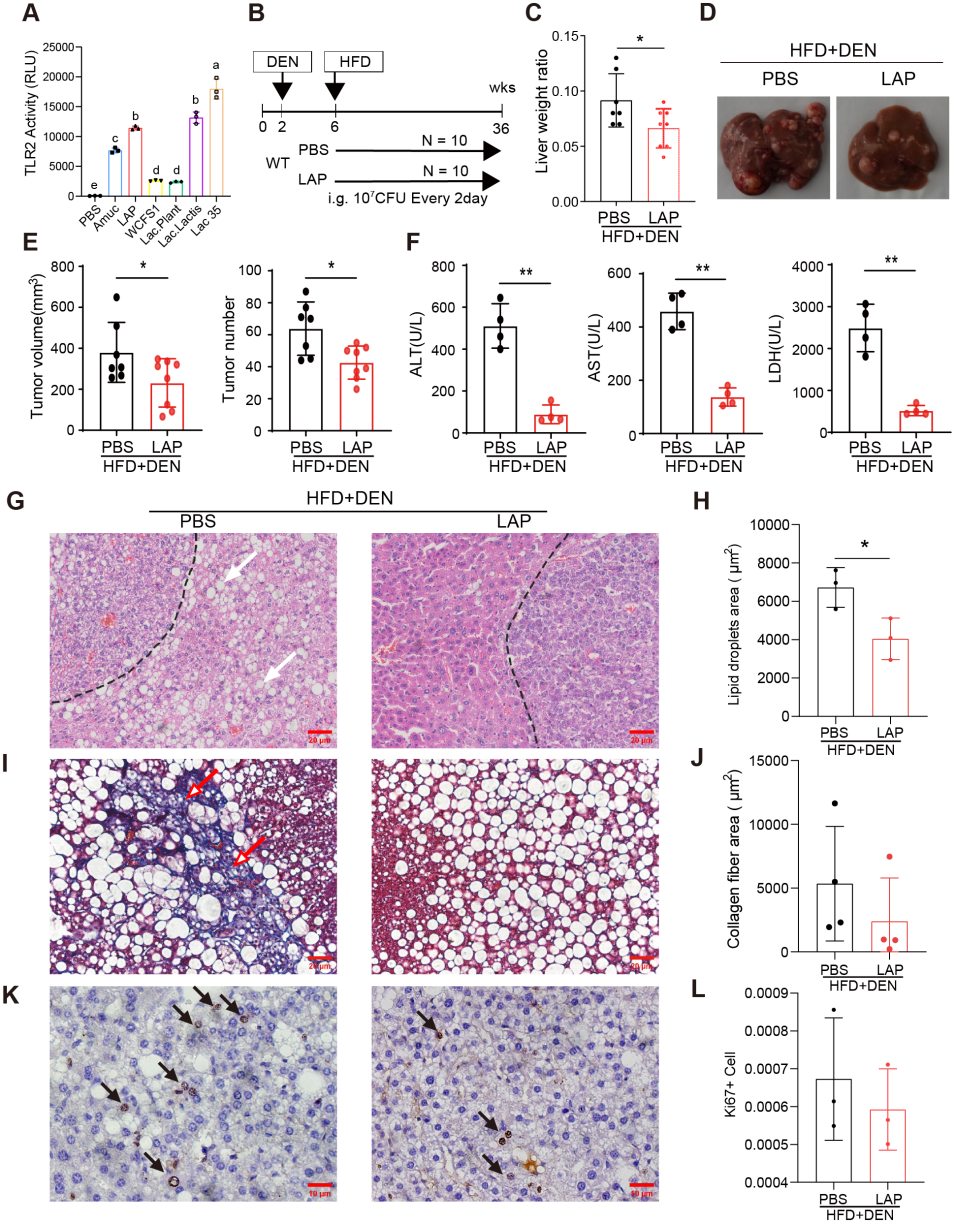
Oral administration of LAP protected mice from DEN-induced liver injury, fibrosis and HCC development. 2-week old male wild-type mice were injected (i.p.) with a single dose of DEN and kept on high fat diet for 36 weeks. Two weeks after DEN injection, mice were divided into two groups and oral garaged with PBS and LAP, respectively. At the endpoint, mouse serum samples and liver tissues were analyzed. **(A)** TLR2 luciferase reporter assay results showing increased TLR2 activity following LAP or individual probiotics treatment *in vitro*. Amuc (a recombinant *Akkermansia muciniphila* membrane protein) was used as a positive control. **(B)** Diagram of treatment timelines. **(C)** Liver-to-body weight ratios of LAP treatment vs control (PBS) groups. **(D)** Representative images of liver from negative control group and DEN-injected mice treated with and without LAP. **(E)** Quantified liver surface tumor numbers and volumes and **(F)** Serum ALT, AST and LDH levels, PBS vs LAP groups. **(G, I, K)** Representative images of H&E **(G)**, Masson’s Trichrome **(I)** and immunohistochemical **(K)** staining showing decreased lipid accumulation (white arrows), fibrosis (red arrows) and Ki67+ proliferating cells (black arrow) in the liver of LAP treated mice. **(H, J, L)** Quantification of lipid droplets and collagen fiber areas in **(F, H)**, and Ki67+ cells in **(J)**, respectively. All data were presented as means ± SD, *, p<0.05; **, p<0.01.

Probiotics are living bacteria that, when administered in adequate amounts, confer a health benefit on the host (41). The broad health benefits of probiotics and their specific effect on cancer suppression have been repeatedly demonstrated in both clinical and experimental settings (42, 43). One mechanism by which probiotics affect host physiology is the stimulation of rDC differentiation (30, 44) in a Toll-like 2 receptor (TLR2) dependent manner (45–47). To develop a probiotics-based approach to reduce hepatic inflammation and to promote liver function, we first conducted a functional screening *in vitro* using a TLR2 activity reporter to identify probiotics that specifically bind to and activate TLR2 signaling. This screening led to the identification of four TLR2-activating lactic acid-producing (LAP) probiotics (**Figure 1A**). To evaluate the potential effects of LAP on hepatic function and homeostasis, we assessed whether LAP-administered mice are protected from or become more sensitive to chemically induced liver injury, fibrosis and HCC formation. For this, LAP was administered once every two days over a 30-week period into mice 4 weeks after intraperitoneal injection of diethylnitrosamine (DEN) (**Figure 1B**). Both control (PBS) and LAP-treated mice were placed on a high-fat diet (HFD) to accelerate tumor growth. Compared to controls, LAP-treated mice had significantly lower liver-to-body weight ratios (**Figure 1C**), reduced numbers and volumes of liver surface tumor (**Figure 1E**) and decreased serum alanine transaminase (ALT), aspartate transferase (AST) and lactate dehydrogenase (LDH) levels (**Figure 1F**). Histological analysis revealed that LAP mice showed markedly reduced hepatic lipid accumulation (**Figures 1G, H**) and fibrosis (**Figures 1I, J**). Notably, the observed hepatoprotective effects LAP were blunted in TLR2 KO mice (**Supplementary Figures S1A–I**).

Next, western blotting and quantitative RT-PCR were performed to assess the hepatic expression of critical genes functionally involved in or regulating cell death (Bcl2, Casp3, Acsl4 and Gpx4), proliferation (Pcna, Ccnd1, Cdk4, p65), inflammation (IL-1β, Il-2, Il-4, Il-6 and Tgfβ) and danger/stress signaling (Tlr2, Tlr4, Tlr5, Tlr9, Rip, Mlkl and P38). None of the listed genes was differentially expressed between the livers of control and LAP mice (**Supplementary Figures S2A–H**). Notably, however, immunohistochemical analysis revealed that LAP mice contained fewer Ki67^+^ cells in their livers than control mice (**Figures 1K, L**). Overall, these findings indicate that mice with expanded hepatic DC subset were protected from DEN-induced liver injury, fibrosis and tumorigenesis, and this hepatoprotective effect was dependent, at least partially, on the TLR2 signaling pathway.

### 3.2 Dietary supplementation of LAP causes accumulation of CD11C^+^ dendritic cells in the mouse liver

**Figure 2:**
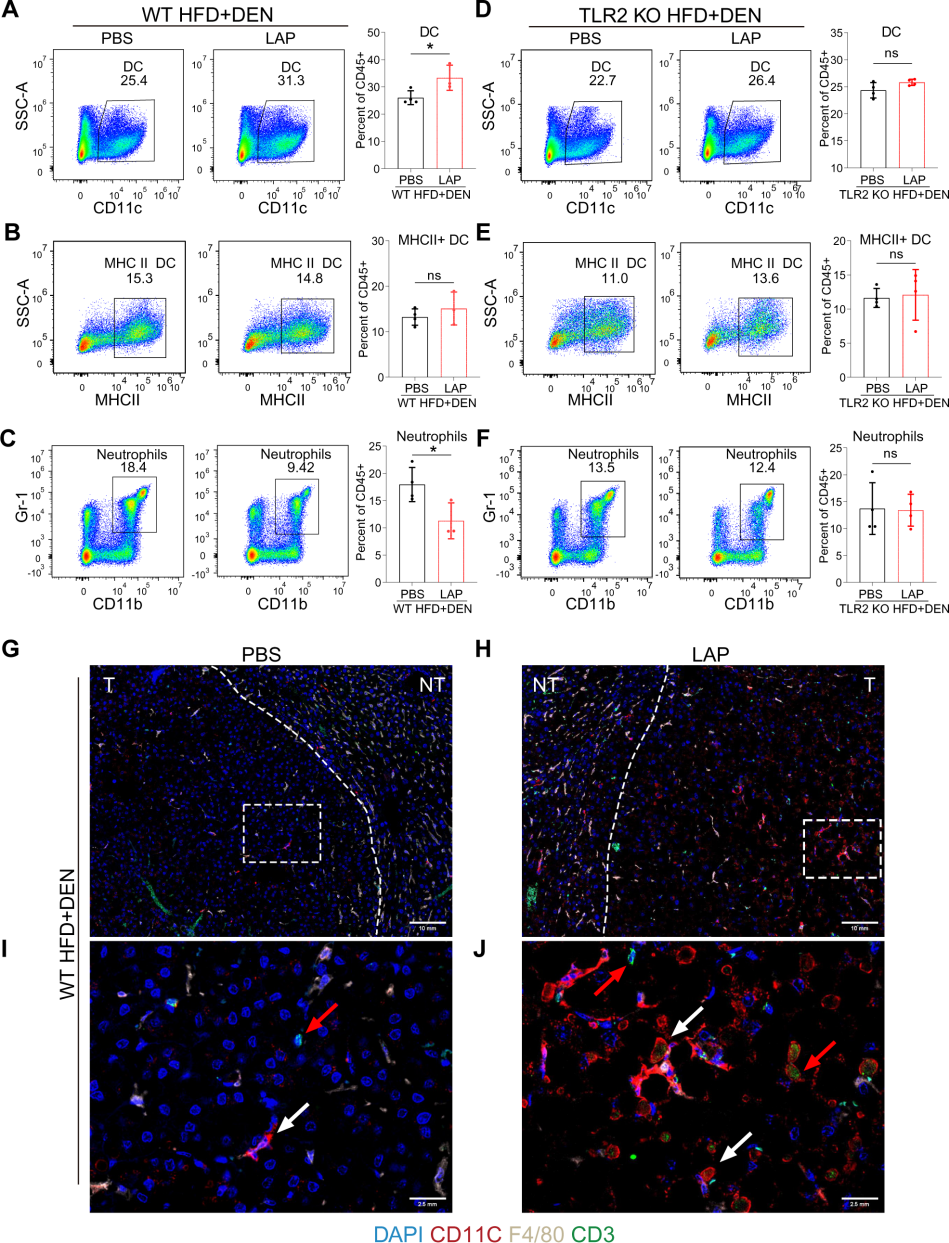
Oral LAP treatment stimulates hepatic dendritic cells in a TLR2-dependent manner. 2-week old male wild-type mice were injected (i.p.) with a single dose of DEN and kept on high fat diet for 36 weeks. Two weeks after DEN injection, mice were divided into two groups and oral garaged with PBS and LAP, respectively. At the endpoint, liver nonparenchymal cells (NPCs) were isolated and analyzed by fluorescence activated cell sorting (FACS) using immune cell type-specific antibodies. **(A–F)** FACS gating strategies and quantifications of percentages of dendritic cells **(A, D)**, MHCII dendritic cells **(B, E)**, neutrophils **(C, F)** in wild-type **(A–C)** and TLR2 knockout **(D–F)** mice treated with PBS or LAP. **(G–J)** Co-immunofluorescence staining showing increased numbers of DCs (white arrows) and T cells (red arrows) in LAP-treated liver. **(I, J)** Magnified images of the dashed squares in **(G)** and **(H)**, respectively. T and NT indicate tumor and non-tumor. All data were presented as means ± SD, *, p<0.05; **, p<0.01; ns means no significant difference.

The liver is populated by a variety of immune cells, including macrophages (Kupffer cells, KC), dendritic cells (DC), natural killer (NK) cells, neutrophils, B and T lymphocytes (11). To determine whether LAP could modulate hepatic immune composition in a TLR2-dependent manner, we analyzed hepatic nonparenchymal cells (NPCs) through fluorescence-activated cell sorting (FACS) (**Supplementary Figure S3**). FACS analysis showed that LAP-treated and control (PBS) mice showed comparable percentages of total immune cells (**Supplementary Figures S4A, G**), macrophages (F4/80^+^) (**Supplementary Figures S4B, H**), myeloid-derived macrophages (MDM) (F4/80^+^/CD11b^+^) (**Supplementary Figures S4C, I**), natural killer cells (NK1.1^+^) (**Supplementary Figures S4D, J**), NKT (CD3^+^NK1.1^+^) (**Supplementary Figures S4E, K**) and total T cells (CD3^+^) (**Supplementary Figures S4F, L**) in the liver. However, LAP-fed WT mice had a significantly higher percentage of dendritic cells (CD11c^+^) (**Figures 2A, G–J**) and lower percentage of neutrophils (Gr-1^+^CD11b^+^) (**Figure 2C**) but no difference in MHCII+ dendritic cells (**Figure 2B**) than control mice. Notably, LAP and PBS-treated WT mice showed no difference in splenic immune composition (**Supplementary Figures S6A–L**), and the observed alterations in hepatic DC and neutrophils were completely blunted in TLR2 KO mice (**Figures 2D–F**; **Supplementary Figures S5A–L**). These results indicate that LAP supplementation did not alter the overall hepatic immune content and landscape of major immune cell types in the liver but selectively affected the proportion of dendritic cells and neutrophils in a TLR2-dependent manner.

### 3.3 LAP-treated mice harbored additional and more subtle immune compositional changes in their hepatic tumor microenvironment (TME)

**Figure 3:**
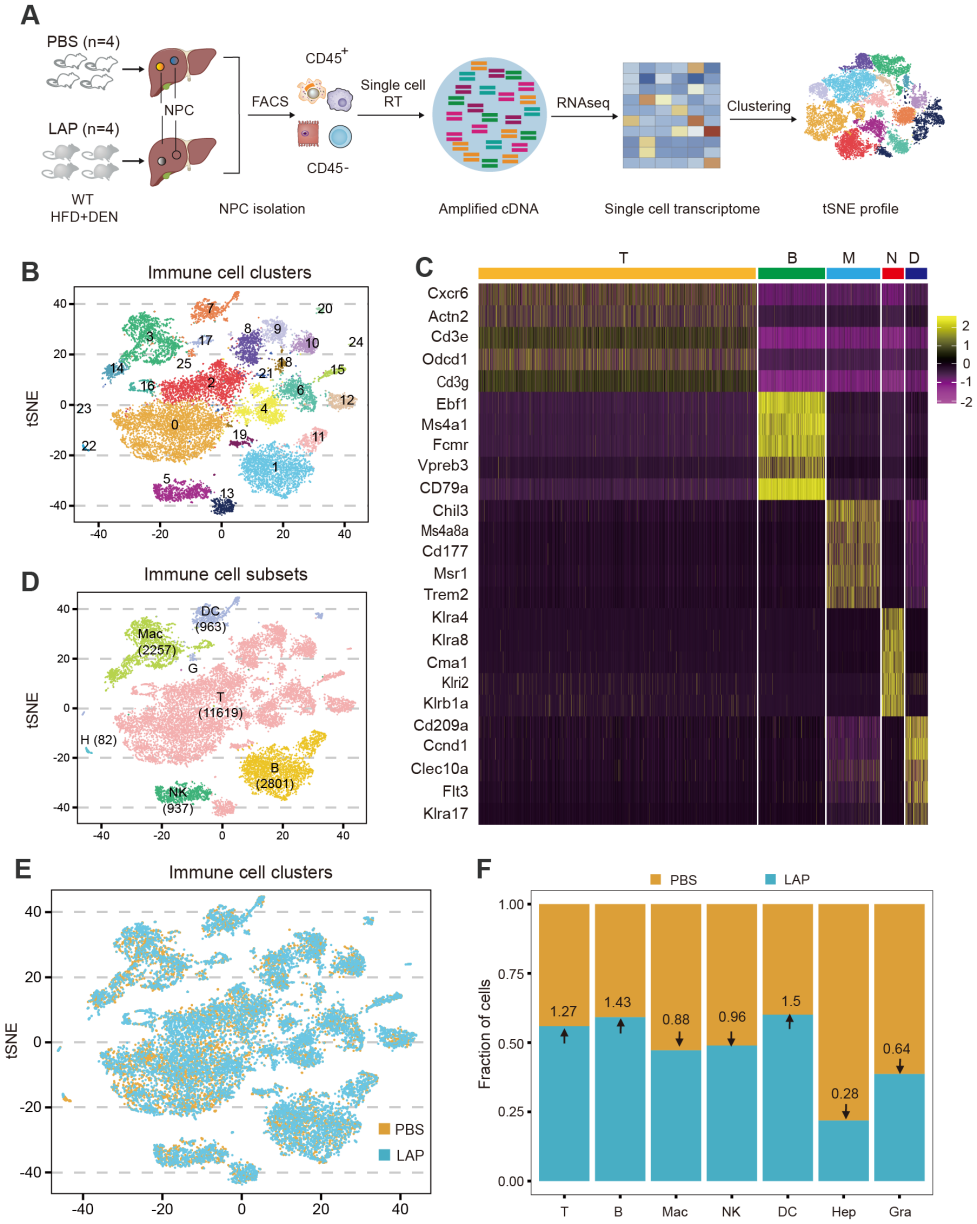
Single-cell transcriptome profiling identifies additional and more subtle LAP-induced changes in hepatic immune composition. Two-week old male wild-type mice were injected (i.p.) with a single dose of DEN and kept on high fat diet treated with PBS or LAP for 36 weeks. At the end point, liver nonparenchymal cells (NPCs) were isolated and subjected to single cell RNA sequencing. **(A)** Diagram of experimental procedures. **(B, D, E)** tSNE plots based on integrated **(B, D)** and separated **(E)** control and LAP group transcriptome data, showing hepatic immune cell clusters **(B)**, defined immune cell subsets **(D)** and differential presence of hepatic immune cell subtypes between control (PBS) vs LAP-treatment **(E)**. **(C)** The heat map shows the enhanced expression of 5 lineage marker genes in T cells, B cells, macrophages, neutrophils, and dendritic cells. **(F)** Calculated fold changes for major immune cell types and hepatocytes, control vs LAP treatment group.

To more quantitatively assess the hepatic immune composition changes induced by LAP, we isolated CD45^+^ cells (total immune cells) from control and LAP-treated mice and performed single-cell RNA sequencing (scRNA-seq) (**Figure 3A**). After quality control of the raw data, a total of 18,690 cells (8392 control and 10298 LAP) were retained, and their single-cell transcriptomic data were used for further analysis. Cell clustering using integrated control and LAP cell data yielded 26 numerically distinct cell subsets (**Figure 3B**). Lineage-specific marker gene-based functional annotation defined these immune cell subsets into 7 functional groups: T cells, B cells, macrophages, natural killer cells, dendritic cells, granulocytes and hepatocytes, with T cells being the most dominant (62%) immune cell type in the liver (**Figures 3C, D**).

Cell clustering analysis was also performed using separated control and LAP cell transcriptomic data to enable a quantitative assessment of the relative abundance of each cell type between control and LAP groups. As shown in **Figures 3E, F**, dendritic cell abundance was increased (1.5-fold), consistent with the FACS data (**Figure 2**), whereas neutrophil abundance was decreased (0.64-fold) in the liver of LAP-treated mice. Notably, the abundance of two other immune cell subtypes, T and B lymphocytes, was also increased (1.27 and 1.43-fold, respectively) in LAP mice. Hence, transcriptome profiling at the single-cell resolution allowed the identification of additional and more subtle immune compositional changes in mice administered with LAP.

### 3.4 Hepatic T cells in LAP-treated mice exhibit increased mitochondrial oxidative phosphorylation but decreased cytotoxic activity

**Figure 4:**
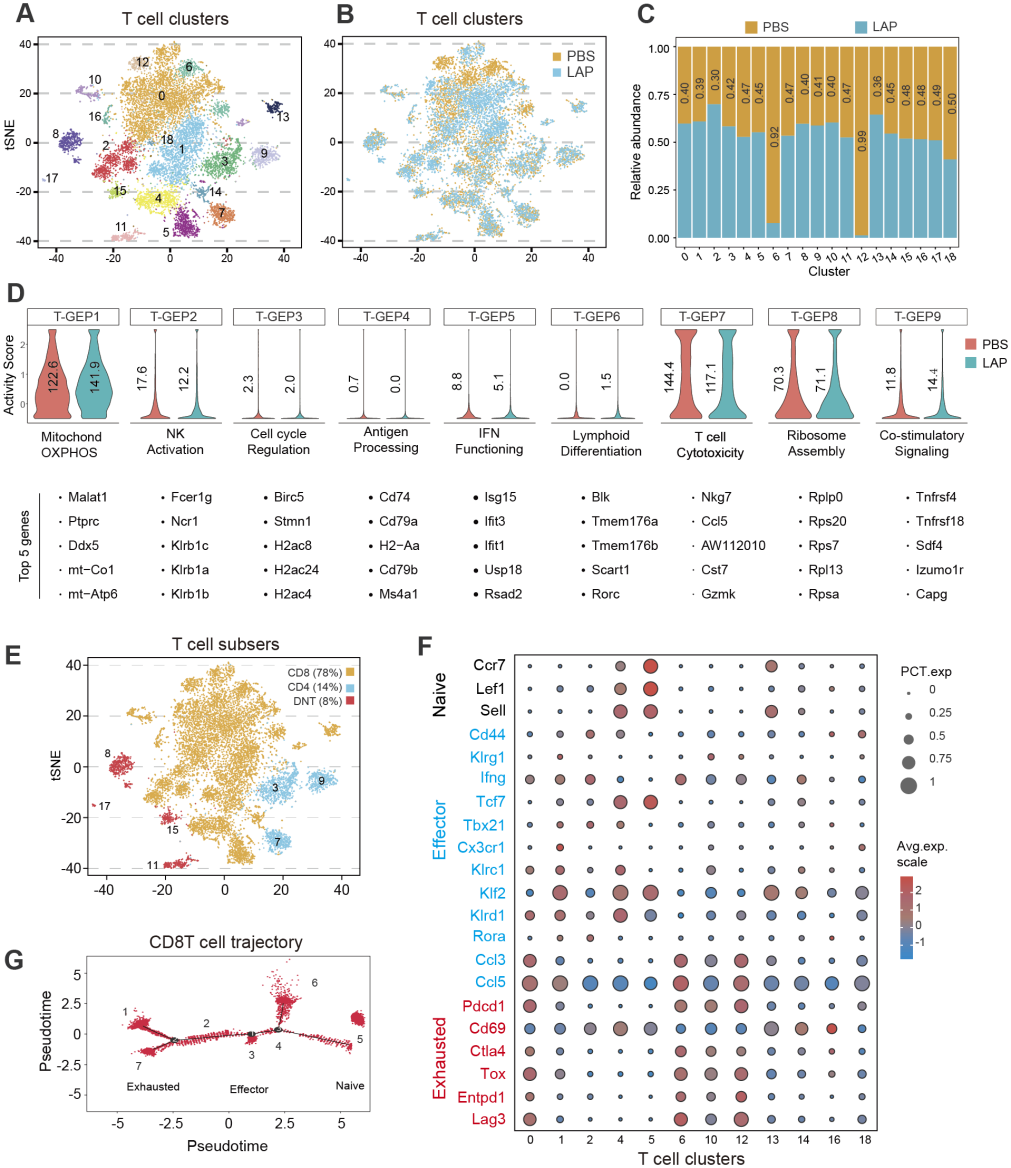
LAP treatment alters the composition and functionality of hepatic T cells. Normalized T cell-specific expression data from control and LAP mice were integrated and used as input to run non-negative factorization (NMF) analysis to identify T clusters of highly similar components inferred as T-GEPs. **(A, B, E)** tSNE plots based on integrated **(A, E)** or separated **(B)** RNAseq data showing various T cell clusters **(A)** and different functional T cell subtypes **(E)**, with differential presence of several T cell clusters between control (PBS) vs LAP treated mice **(B)**. **(C)** Bar graph showing differentially present hepatic immune cell subtypes between control (PBS) vs LAP-treatment. Horizontal numbers indicate cluster-specific cell number fold changes. **(D)** Violin plots showing altered T cell-specific gene expression programs (T-GEPs) between control and LAP mice. Vertical numbers indicate GEP activity score. Top 5 of the 100 weighted genes were listed below each GEP. **(F)** Bubble chart showing differential expression of naïve, effector and exhausted markers between the identified T cell clusters. **(G)** Marker gene-based trajectory analysis for the CD8T subset.

Because T cells were dominantly present in the TME of DEN-induced liver tumors (**Figures 3C, D**) and have been recognized as the significant cytotoxic immune cells (48, 49), we further analyzed the compositional and functional changes of the T cell subset resulting from LAP treatment. Cell clustering analysis using integrated control and LAP cell transcriptome data yielded 19 T cell clusters (**Figure 4A**), which were further defined into three functional sub-types: CD8^+^T, CD4^+^T and double-negative T (DNT) (**Figure 4E**), based on marker gene expression. Cell clustering using separated control and LAP cell transcriptomic data revealed that clusters 6 and 12 (C06 and C12) were significantly diminished, whereas other T cell clusters were modestly expanded in the LAP group (**Figures 4B, C**). Notably, both C06 and C12 showed high expression of Pdcd1, Cd69, Ctla4, Tox, Entpd1 and Lag3, markers of exhausted T cells, as well as Ccl3 and Ccl5, genes functionally associated with effector T-cell function (50) (**Figure 4F**). Other clusters showed high expression of either effector or naïve T cell markers or a mixed expression of both types of markers (**Figure 4F**).

To understand how LAP treatment affected the functionality of T cells, consensus non-negative matrix factorization (cNMF), a novel algorithm developed to more accurately infer identity versus activity program (38), was used to establish gene expression programs (GEPs). cNMF analysis yielded 9 T cell-specific GEPs (T-GFPs) (**Figure 4D**). Based on functional annotation of the top 30 differentially expressed genes, these T-GFPs controlled nine distinct functional pathways: mitochondrial oxidative phosphorylation (OXPHOS) (T-GEP1), natural killer cell activation (T-GEP2), cell cycle activation (T-GEP3), antigen presentation (T-GEP4), interferon-gamma functioning (T-GEP5), lymphoid differentiation (T-GEP6), T cell cytotoxicity (T-GEP7), ribosome assembly (T-GEP8) and co-stimulatory signaling (T-GEP9) (**Figure 4D**). LAP treatment, while increasing the activity of lymphocyte mitochondrial OXPHOS (T-GEP1) and co-stimulatory signaling (T-GEP9), notably decreased the activity of NK activation (T-GEP2), IFN functioning (T-GEP 5) and T cell cytotoxicity (T-GEP7). Furthermore, marker gene-based trajectory analysis for the CD8T subset and various T-cell clusters indicated that LAP feeding did not affect the differentiation of effector and exhausted T cells from naïve T progenitors (**Figure 4G**, **Supplementary Figure S8A**).

### 3.5 Oral supplementation of LAP induces regulatory dendritic cells in the liver

**Figure 5:**
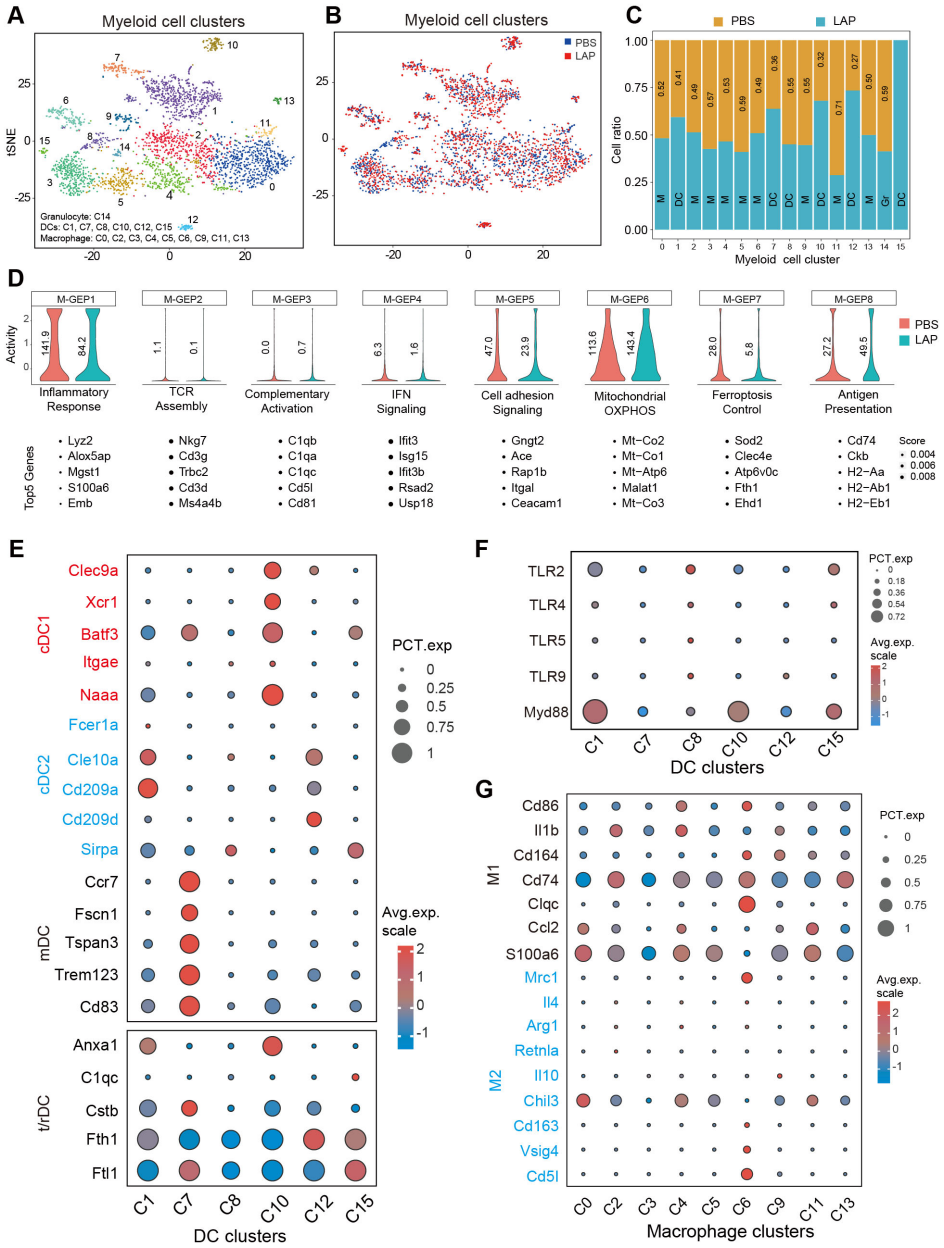
LAP treatment induces regulatory/tolerogenic dendritic cells in the liver. Normalized myeloid cell-specific expression data from control and LAP mice were integrated and used as input to run non-negative factorization (NMF) analysis to identify myeloid clusters of highly similar components inferred as M-GEPs. **(A, B)** tSNE plots of various myeloid cell clusters based on integrated **(A)** and separated **(B)** RNAseq data. **(C)** Bar graph showing differentially present hepatic immune cell subtypes between control (PBS) vs LAP-treatment. **(D)** Violin plots showing altered myeloid cell-specific gene expression programs (M-GEPs) between control and LAP mice. Vertical numbers indicate GEP activity score. Top 5 of the 100 weighted genes were listed below each GEP. **(E–G)** Bubble charts revealing differential expression of cDC1, cDC2, mDC and r/tDC, and M1 and M2-like macrophage markers **(E, G)** and TLR signaling related genes between the identified myeloid cell clusters.

As first immune responders, myeloid cells (macrophages, dendritic cells, monocytes and granulocytes) sense infection or tissue damage and direct the recruitment, proliferation and activation of adaptive immune cells (51). Hence, we wondered whether compositional and functional alterations of the myeloid compartment caused the observed hepatic T cell activity and functional changes in LAP-treated mice. Clustering analysis showed that the hepatic myeloid compartment was composed of 16 distinct clusters, which were further defined into 3 functional groups: granulocytes, dendritic cells and macrophages, by marker gene expression analysis (**Figure 5A**). LAP administration increased the abundance of C01, C07, C10, C12 and C15, while decreased the abundance of C03, C05, C11 and C14 (**Figures 5B, C**). Notably, all clusters expanded in the LAP group were dendritic cells expressing two or more regulatory or tolerogenic dendritic cell markers (**Figure 5E**), such as Anxa1, C1qc, Cstb and Fth1 (52). Interestingly, these DC clusters all express TLR2 and its downstream adaptor Myd88 (**Figure 5F**). On the contrary, the unchanged or diminished clusters in the LAP group were either macrophages or granulocytes that expressed two or more M1-type macrophage markers (**Figure 5G**), such as Cd86, Il1b, Cd164, Cd74, Clqc, Ccl2 and S100a6 (53). These findings support the notion that LAP supplementation induces regulatory or tolerogenic dendritic cells in the liver.

Next, gene expression programs (GEPs) of hepatic myeloid cells were calculated and compared between the control and LAP groups. cNMF analysis produced 8 myeloid-specific GEPs (M-GEP1 to M-GEP8) (**Figure 5D**). These M-GEPs control eight distinct myeloid functional pathways, including inflammatory response (M-GEP1), TCR assembly (M-GEP2), complementary activation (M-GEP3), IFN signaling (M-GEP4), cell adhesion signaling ((M-GEP5), mitochondrial OXPHOS (M-GEP6), ferroptosis control (M-GEP7) and antigen presentation (M-GEP8) (**Figure 5D**). LAP treatment increased the activity of myeloid cell mitochondrial OXPHOS (M-GEP6) and antigen presentation (M-GEP8) while decreasing the activity of inflammatory response (M-GEP1), IFN signaling (M-GEP4), cell adhesion signaling (M-GEP5) and ferroptosis (M-GEP7) (**Figure 5D**). We re-examined the functions of the top 30 differentially expressed genes in M-GEP6 through Reactome pathway analysis and found that they were enriched in bioenergetic functions, such as cellular respiratory electron transport, ATP synthesis and TCA cycle (**Supplementary Figures S8B, C**). Collectively, these results suggest that oral administration of LAP induces regulatory or tolerogenic dendritic cells that promote an immunosuppressive tumor microenvironment in the liver.

## 4 Discussion

DCs are versatile antigen-presenting cells with essential roles in the initiation and regulation of “*danger*”-specific T cell responses. Thus, DCs have long been considered an attractive drug target for immune-based treatment of liver diseases (54, 55). Despite this, few clinical benefits of DC-based therapy have been demonstrated thus far, in part due to the lack of efficient DC-modulating reagents. Here, we demonstrate that LAP, a novel mix of TLR2-interacting and lactic acid-producing probiotics, are potent promoters of hepatic DCs. Mice orally administered with LAP had significantly higher numbers of DCs in the liver and were protected from diethylnitrosamine-induced liver injury, fibrosis and tumorigenesis, in a TLR2-dependent manner. Single-cell transcriptome profiling revealed that the hepatic T cells of LAP-treated mice exhibit enhanced mitochondrial oxidative phosphorylation but reduced cytotoxicity activity. LAP treatment increased mitochondrial oxidative phosphorylation and antigen presentation activities while decreasing the inflammatory response of hepatic myeloid cells. The observed LAP-responsive DCs in the liver expressed two or more regulatory or tolerogenic markers. Collectively, our data suggest that the TLR2-activating probiotics identified in the current study are potent promoters of hepatic regulatory dendritic cells and can thus be utilized to devise probiotics-based approaches for effective protection of the liver against toxin or metabolic stress-induced hepatocellular damages and tumorigenesis.

Diverse innate and adaptive immune cells, including macrophages, dendritic cells polymorphonuclear neutrophils (PMN) and lymphocytes express one family of 13 receptors, the toll-like receptors (TLRs) that recognize pathogen-associated molecular patterns (PAMPs) or endogenous danger-associated molecule patterns (DAMPs) (56). The recognition of PAMPs and/or DAMPs by different TLRs triggers distinct signaling pathways, including NF-kB, p38, JNK and ERK, causing upregulation of proinflammatory genes and immune reactions (57). Of note, one member of the TLR family, TLR2, has been shown to play a unique immune modulatory role by recognizing probiotics or other intestinal commensals to elicit immuno-suppressive action (58). In line with previously reported data, we find in this study that LAP administration lowers DEN-induced liver injury in WT but not in TLR2 KO mice (**Figures 1C–F**; **Supplementary Figures S1B–I**). In addition, LAP stimulated modest upregulation of immunosuppressive cytokines IL-4 and IL-10 in WT but not in TLR2 KO mice (**Supplementary Figures S2A–D**). Moreover, single cell transcriptomic profiling indicates that both TLR2 and its downstream signaling adaptor Myd88 are highly expressed in the LAP-stimulated DC clusters (**Figure 5F**). Together, these results suggest that one mechanism by which LAP elicits immunosuppressive effects is to bind and activate the TLR2 signaling pathway.

Dendritic cells are developmentally and functionally heterogeneous. Depending on the nature of the stimulating cues, DCs, which are commonly classified into conventional (cDC), plasmacytoid (pDC) and monocyte-derived (mDC), can either promote (immunogenic) or suppress (tolerogenic or regulatory) tissue inflammation (59). Through single-cell transcriptome profiling, we identified seven hepatic DC clusters. Compared to other defined myeloid cell clusters (macrophages and granulocytes), all DC clusters consistently showed high mRNA expression of Flt3 and Batf3, critical regulators of monocytes to DCs differentiation (60, 61). These findings indicate that the DC clusters observed here were likely derived from monocytes and may function as APCs to activate CD8^+^T cells, much as the CD103^+^ DCs observed in other tissue microenvironments (62). It is noteworthy that oral LAP administration in mice increased the hepatic abundance of five but one DC cluster and that all expanded DCs show high mRNA expression of one or more regulatory or tolerogenic DC markers (also known as DC3) (52, 63). More importantly, gene expression program (GEP) activity analysis revealed that LAP treatment broadly decreased hepatic T cell functionality, including reduction of the inflammatory response, downregulation of IFN signaling and cytotoxicity. Based on these findings, we suggest that the LAP-responsive hepatic DCs observed in our study are regulatory or tolerogenic DCs that may directly interact with hepatic T cells to suppress immunogenicity.

Oral administration of LAP markedly diminished two hepatic T cell clusters (C06 and C12) while modestly expanded several others (**Figure 4C**). We wondered whether the two diminished T cell clusters are Th1, Th2 or Th17 helper cells, as these cells are proinflammatory and are readily inducible by probiotics treatment (30). Notably, both C06 and C12 are transcriptionally positive for cytotoxic T lymphocyte markers, including Cd8, Gzmk, Gzma, Gnly and Gzmb (64, 65), but negative for Il-17, a potent proinflammatory cytokine secreted by T helper 17 (Th17) T cells (66, 67). Furthermore, C06 and C12 show higher transcription of several exhausted (Pdcd1, Cd69, Ctla4, Tox, Entpd1 and Lag3) and effector (Ccl3, Ccl5 and Nkg7) T cell markers (50, 68, 69). Based on these findings, we speculate that C06 and C12 represent two immune-reactive effector CD8^+^ T cells that are undergoing rDC-mediated T cell exhaustion. These cells are highly inflammatory but molecularly different from the IL-17-producing CD4^+^ T helper cells, whose abundance was reportedly decreased by probiotics treatment (31). Further studies are needed to shed light on the cellular and immunological features of these cells and how they interact with antigen-presenting cells during immune coordination.

Another intriguing finding of the current study is that oral administration of LAP in mice significantly increases mitochondrial oxidative phosphorylation in both myeloid cells and T lymphocytes. This suggests that LAP supplementation may benefit innate and adaptive immune cell health by increasing mitochondrial functionality. Presently, the mechanisms underlying the LAP-mediated metabolic upregulation remain unclear. From a therapeutic or prophylactic perspective, this may be useful for improving future cancer immunotherapy. In the solid tumor microenvironment (TME), rapidly proliferating cancer cells compete, often disproportionally, with tumor-infiltrating immune cells for glucose and other nutrients (70). The decreased nutrient contents impose metabolic stress on and impair the function of immune cells, resulting in rapid tumor growth (71). Restoring nutrient supply to or reprogramming metabolic requirements of tumor infiltrating immune cells are potential strategies that can be used clinically to reverse premature immune cell exhaustion and to increase the success of immunotherapy (72, 73). In this direction, further studies are needed to test the safety and efficacy of LAP as well as other immunogenic probiotics as an immune checkpoint blockade therapy adjuvant in both preclinical models and in clinical settings. Finally, given the characterized role of LAP in stimulating regulatory DCs, we anticipate that it will have several other important clinical applications, including the prevention or treatment of autoimmune disorders (arthritis and asthma), inflammatory bowel disease and alcohol/nonalcohol-induced chronic liver diseases.

## Data Availability

The raw sequence data reported in this paper have been deposited in the Genome Sequence Archive in National Genomics Data Center, China National Center for Bioinformation / Beijing Institute of Genomics, Chinese Academy of Sciences (GSA: CRA024153) that are publicly accessible at https://ngdc.cncb.ac.cn/gsa.
