# Supplementary material for: Stimulation of regulatory dendritic cells suppresses cytotoxic T cell function and alleviates DEN-induced liver injury, fibrosis and hepatocellular carcinoma
Source: Front Immunol. 2025 Apr 8;16:1565486. doi: 10.3389/fimmu.2025.1565486 (PMC12011597; doi:10.3389/fimmu.2025.1565486)

**Supplementary Figure Legends**

**Figure S1 LAP-mediated hepatoprotective effects were blunted in TLR2 knockout mice.** 2-week old male TLR2 KO mice were injected (i.p.) with a single dose of DEN and kept on high fat diet for 36 weeks. Two weeks after DEN injection, mice were divided into two groups and oral garaged with PBS and LAP, respectively. At the endpoint, mouse serum samples were analyzed. (A) Diagram showing experimental procedure**.** (B) Representative images of liver from DEN-injected mice treated with and without LAP. Arrows indicate surface tumors. (C-D) Quantified liver surface tumor numbers (C) and volumes (D) between control and treatment groups. (E-I) Quantified liver-to-body weight ratio (E), serum ALT (F), AST (G), ALP (H) and LDH (I) levels of control vs treatment groups. N.S., not significant.

**Figure S2 LAP administration shows little impact on hepatic expression of cell proliferation, death and proinflammatory markers.** 2-week old male WT and TLR2 KO mice were injected (i.p.) with a single dose of DEN and kept on high fat diet for 36 weeks. Two weeks after DEN injection, mice were divided into two groups and oral garaged with PBS and LAP, respectively. At the endpoint, liver RNA and protein were prepared for qRT-PCR and Western blotting. **(A-D)** Quantified mRNA expression of anti- **(A, C)** and pro- **(B, D)** inflammatory cytokine genes in WT (A-B) and TLR2 KO (C-D). **(E-H)** Representative Western blotting images showing unaltered expression of TLR receptors **(E)**, cell death regulators **(F)**, stress-responsive factors **(G)** and cell cycle regulators **(H)** between control and LAP-treated mice.

**Figure S3：Gating strategy used for FACS analysis of hepatic immune cells in WT and TLR2 KO mice.** FSC-A and FSC-H were used to exclude cell aggregates and Fixable Viability was used to identify dead cells. Stained cells were gated on immune cells based on the SSC-A and CD45, and subsequently, defined using the following surface markers: DC (CD11c+); MHC Ⅱ DC (CD11c+, MHC Ⅱ+); Macrophage (F4/80+); CD11b Macrophage (F4/80+, CD11b+); B cell (CD19+, CD3-); T cell (CD19-, CD3+); Neutrophil (CD19-, CD3-, Gr1+, CD11b+); NK (CD19-, CD3-, NK1.1+); NK T (CD19-, CD3+, NK1.1+)

**Figure S4 FACS detected no significant difference in other immune cell types between control and LAP-treated WT mice.** 2-week old male WT mice were injected (i.p.) with a single dose of DEN and kept on high fat diet for 36 weeks. Two weeks after DEN injection, mice were divided into two groups and oral garaged with PBS and LAP, respectively. At the endpoint, hepatic immune cells were analyzed by FACS. **(A-F)** Gating strategies used in FACS analysis of hepatic total immune cells **(A)**, macrophages **(B)**, monocyte-derived macrophages **(C)**, natural killer cells **(D)**, natural killer T cells **(E)**, and B and T cells **(F)**. **(G-L)** Quantification of percentages of total immune cells **(G)**, macrophages **(H)**, monocyte-derived macrophages **(I)**, natural killer cells **(J)**, natural killer T cells **(K)**, B and T cells **(L)**. N.S., not significant.

**Figure S5 LAP-mediated immune-modulating effects were blunted in TLR2 KO mice.** 2-week old male TLR2 KO mice were injected (i.p.) with a single dose of DEN and kept on high fat diet for 36 weeks. Two weeks after DEN injection, mice were divided into two groups and oral garaged with PBS and LAP, respectively. At the endpoint, hepatic immune cells were analyzed by FACS. **(A-F)** Gating strategies used in FACS analysis of hepatic total immune cells **(A)**, macrophages **(B)**, monocyte-derived macrophages **(C)**, natural killer cells **(D)**, natural killer T cells **(E)**, and B and T cells **(F)**. **(G-L)** Quantification of percentages of total immune cells **(G)**, macrophages **(H)**, monocyte-derived macrophages **(I)**, natural killer cells **(J)**, natural killer T cells **(K)**, B and T cells **(L)**. N.S., not significant.

**Figure S6 LAP treatment does not alter splenic immune cell composition and function.** 2-week old male WT mice were injected (i.p.) with a single dose of DEN and kept on high fat diet for 36 weeks. Two weeks after DEN injection, mice were divided into two groups and oral garaged with PBS and LAP, respectively. At the endpoint,splenocytes were prepared and analyzed by FACS. **(A)**, T cells **(B)**, T/B cell ratio **(C)**, CD4 T cells **(D)**, CD8 T cells **(E)**, dendritic cells **(F)**, MHC II+ dendritic cells **(G)**, macrophages **(H)**,monocyte-derived macrophages (MDM) **(I)**, natural killer cells **(J)**, natural killer T cells **(K)** and neutrophils **(L)**. N.S., not significant.

**Figure S7: The gut microbiota difference between WT and TLR2 KO mice is insufficient to impact DEN-induced liver tumor formation and growth.** The feces of TLR2 KO mice and their littermate control mice were collected and subjected to 16S RNA sequencing. For fecal microbiota transplantation (FMT), 2-week-old WT mice were injected (i.p.) with a single dose of DEN and orally received an antibiotic cocktail for a month starting at 6 weeks of age. Subsequently, the mice were divided into two groups, receiving fecal suspension for 6 months from WT (FMT-WT) and TLR2 KO (FMT-KO) mice, respectively. **(A-D)** Quantified alpha (A-C) and beta (D-E) diversities of WT and TLR2 KO mice. **(F)** Diagram showing the experimental design of fecal microbiota transplantation. **(G)** Representative images of liver from DEN-injected mice after FMT treatment. **(H-J)** Quantified liver-to-body weight ratios **(H)**, liver surface tumor numbers **(I)** and maximum tumor volume **(J)** of FMT-KO vs FMT-WT groups. NS indicates nonsignificant.

**Figure S8 LAP treatment does not impair T cells maturation but increases myeloid cells mitochondrial oxidative phosphorylation**. 2-week old male wild-type mice were injected (i.p.) with a single dose of DEN and kept on high fat diet treated with PBS or LAP for 36 weeks. At the end point, liver nonparenchymal cells (NPCs) were isolated and subjected to single cell RNA sequencing. **(A)** Single-cell transcriptome-based trajectory analysis showing normal maturation of hepatic naïve T cells in control and LAP-treated mice. **(B-C)** Heatmap **(B)** and bubble chart **(C)** showing differentially expressed top 30 genes between control and treatment groups and their enriched pathways.


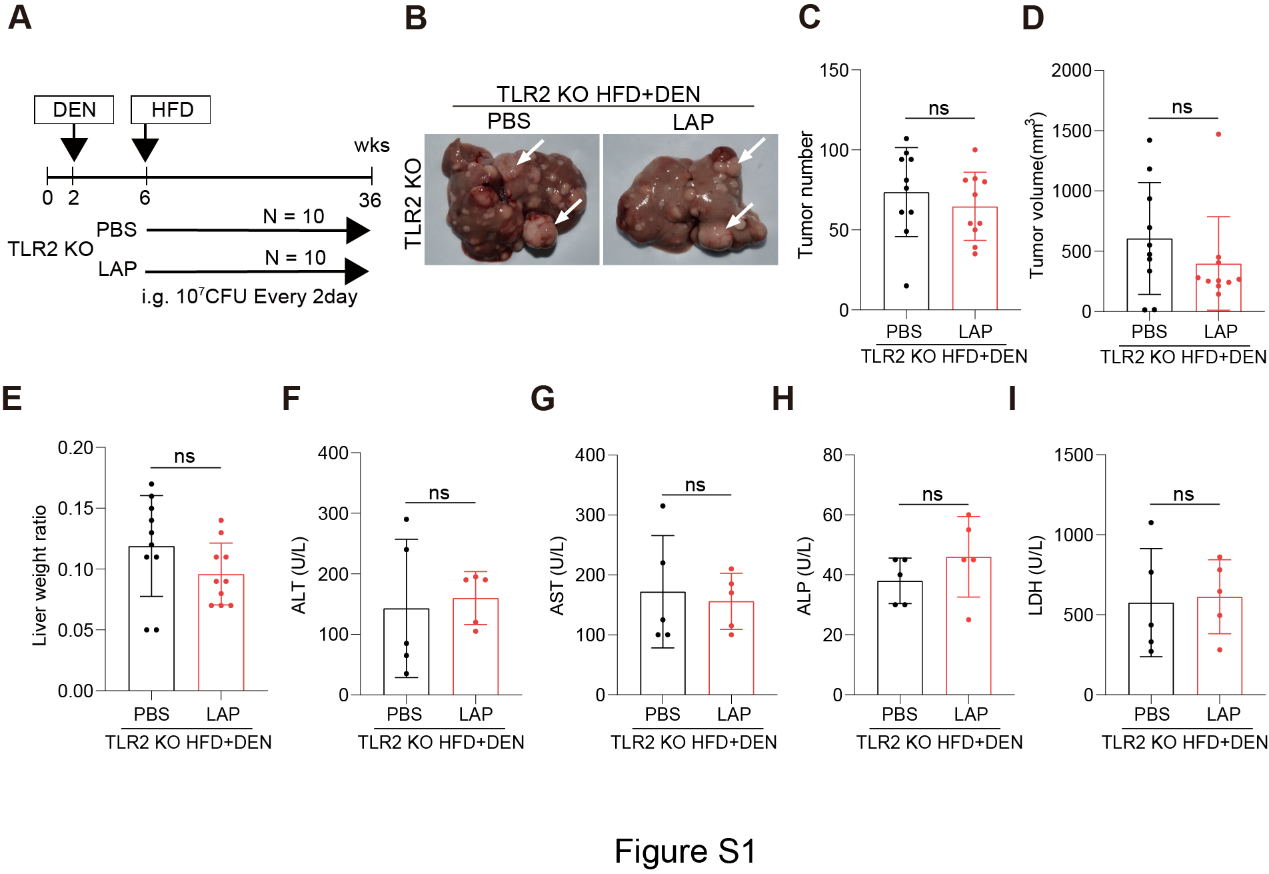


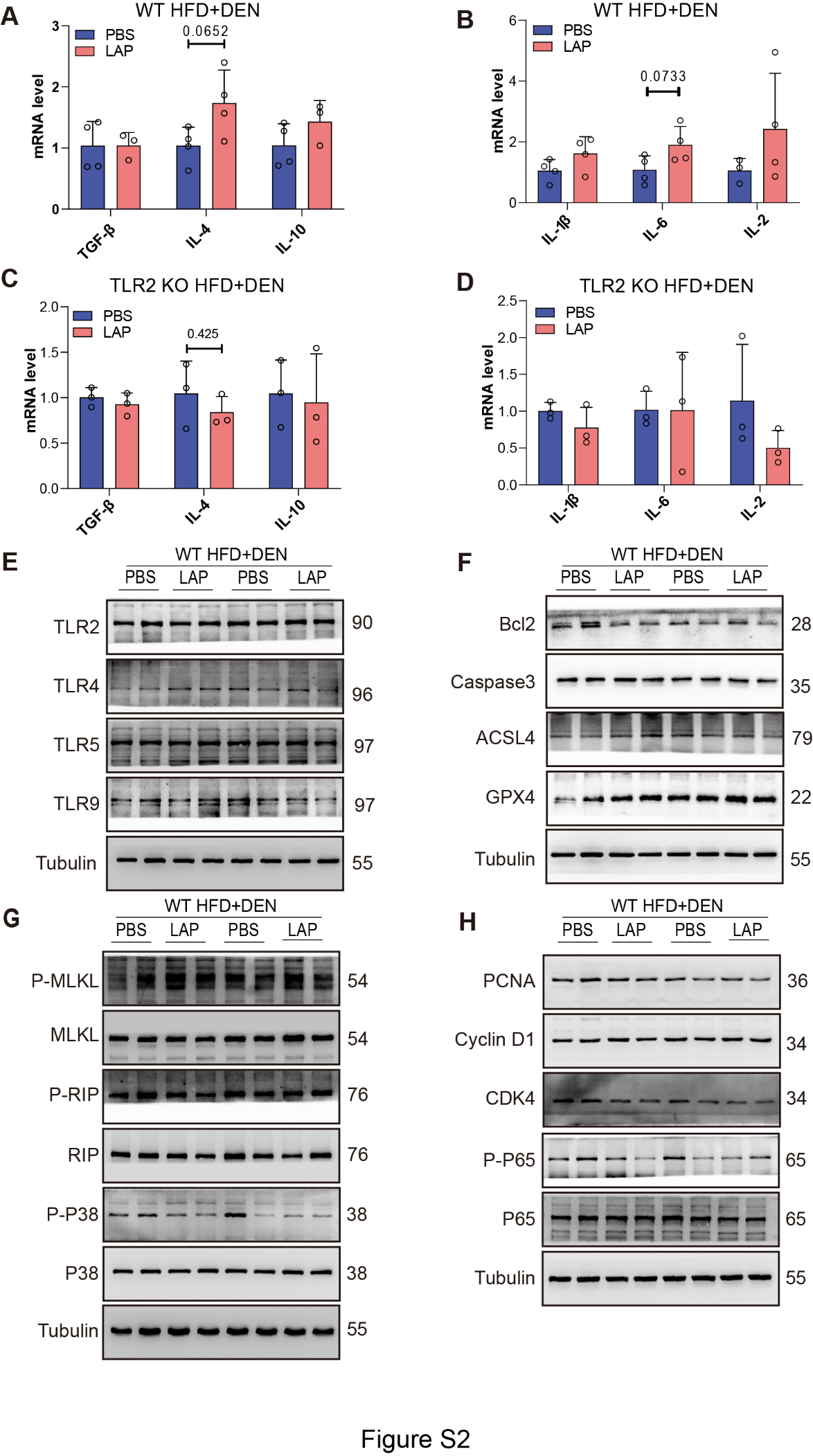


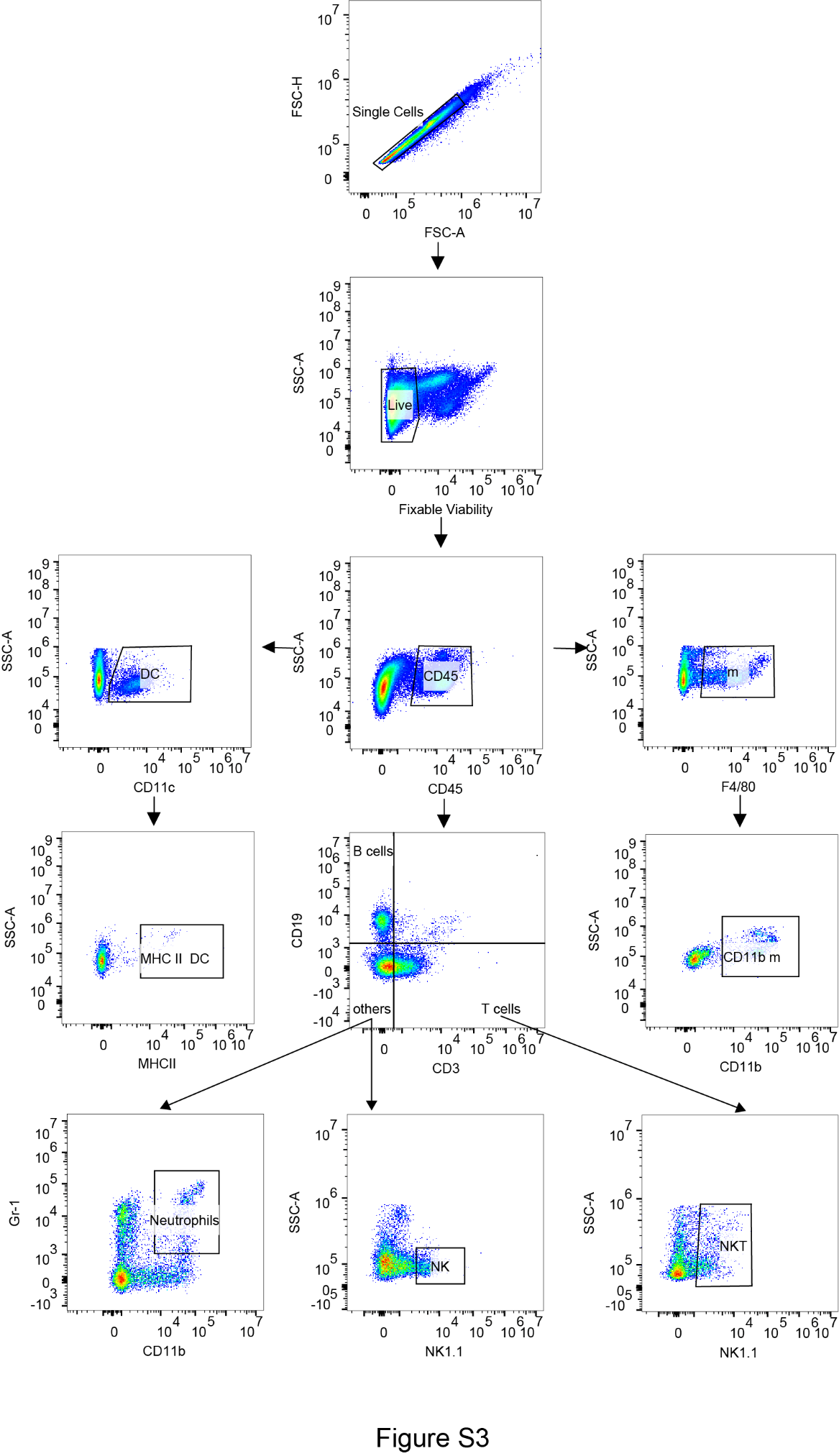


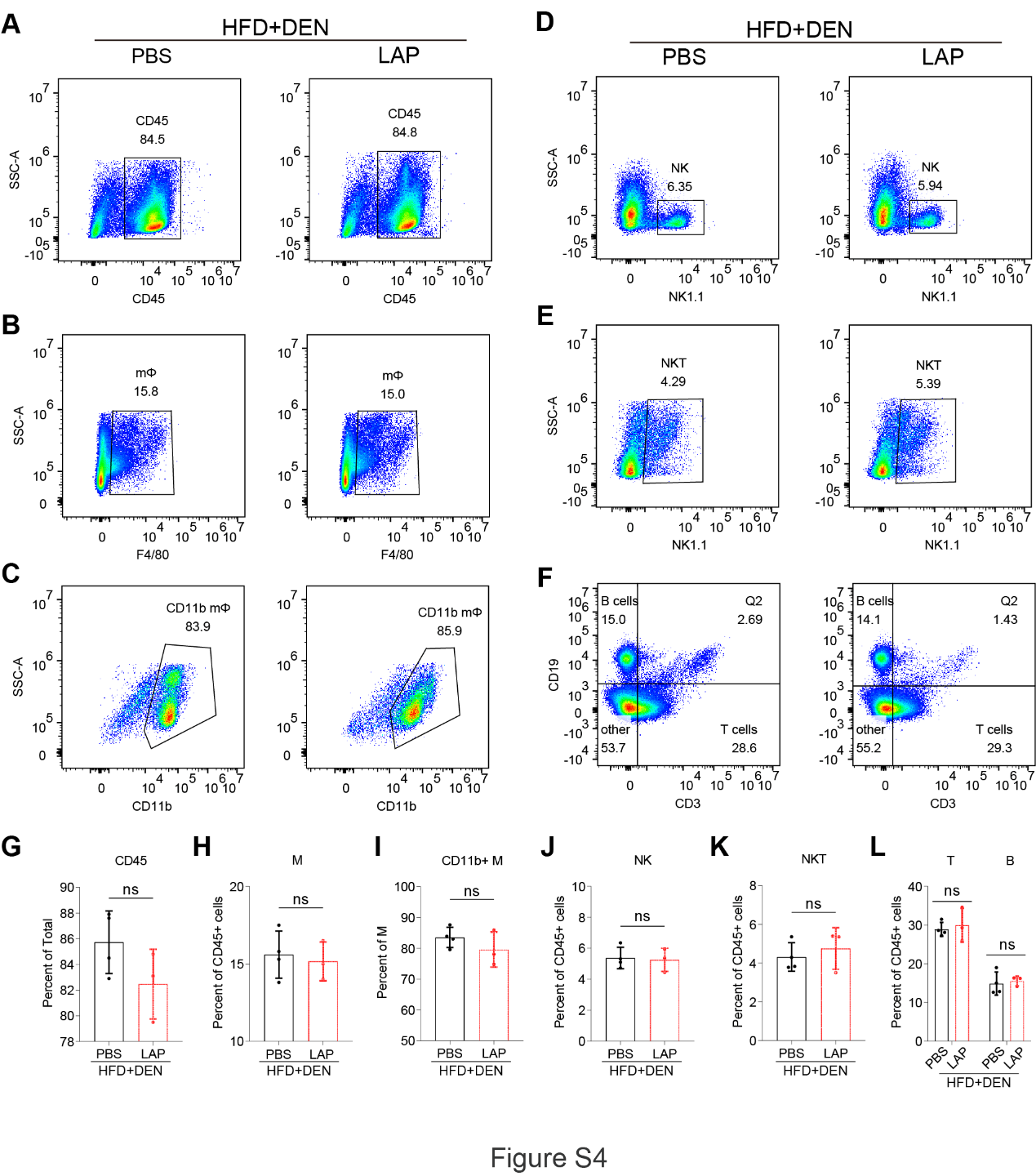


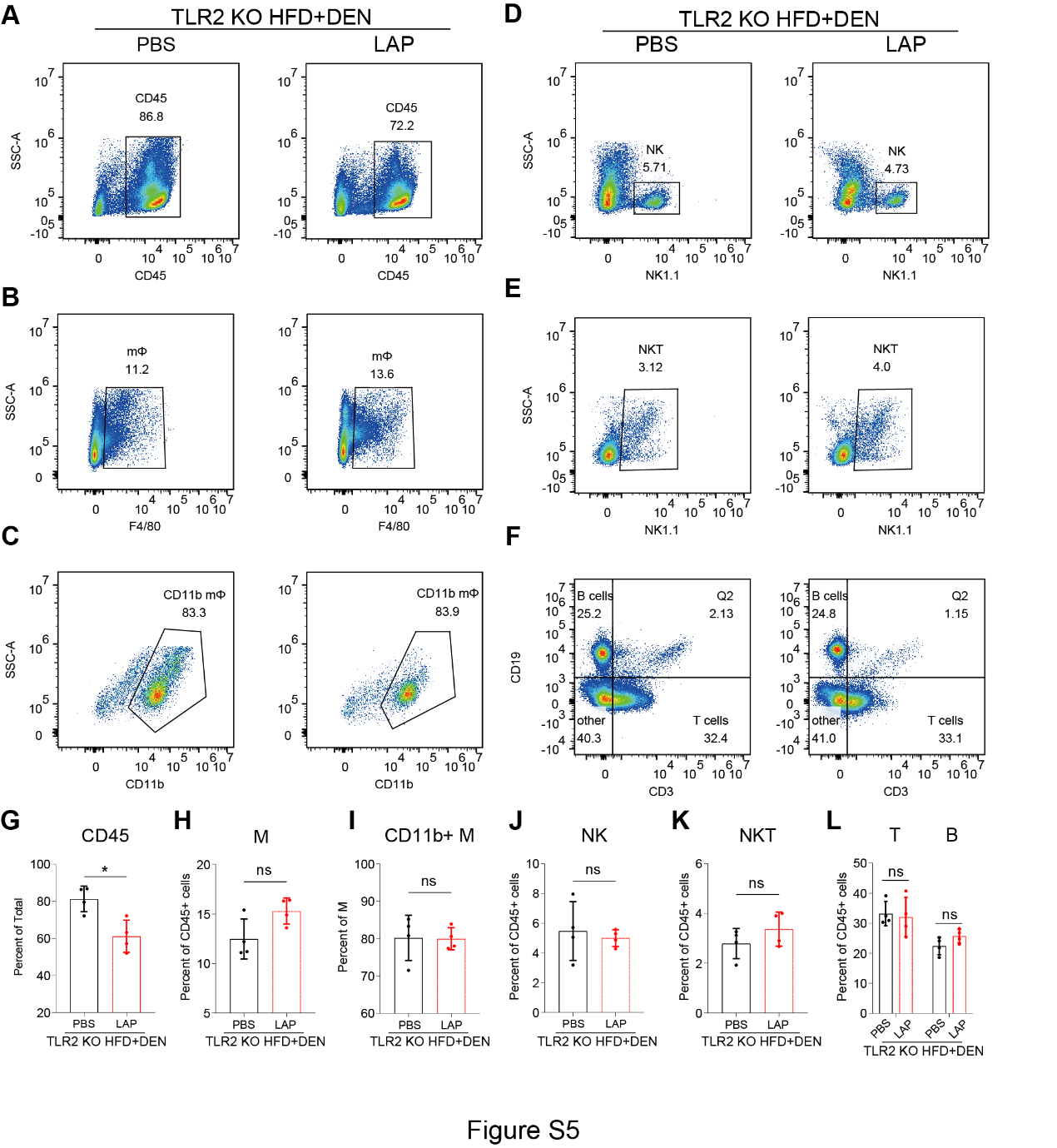


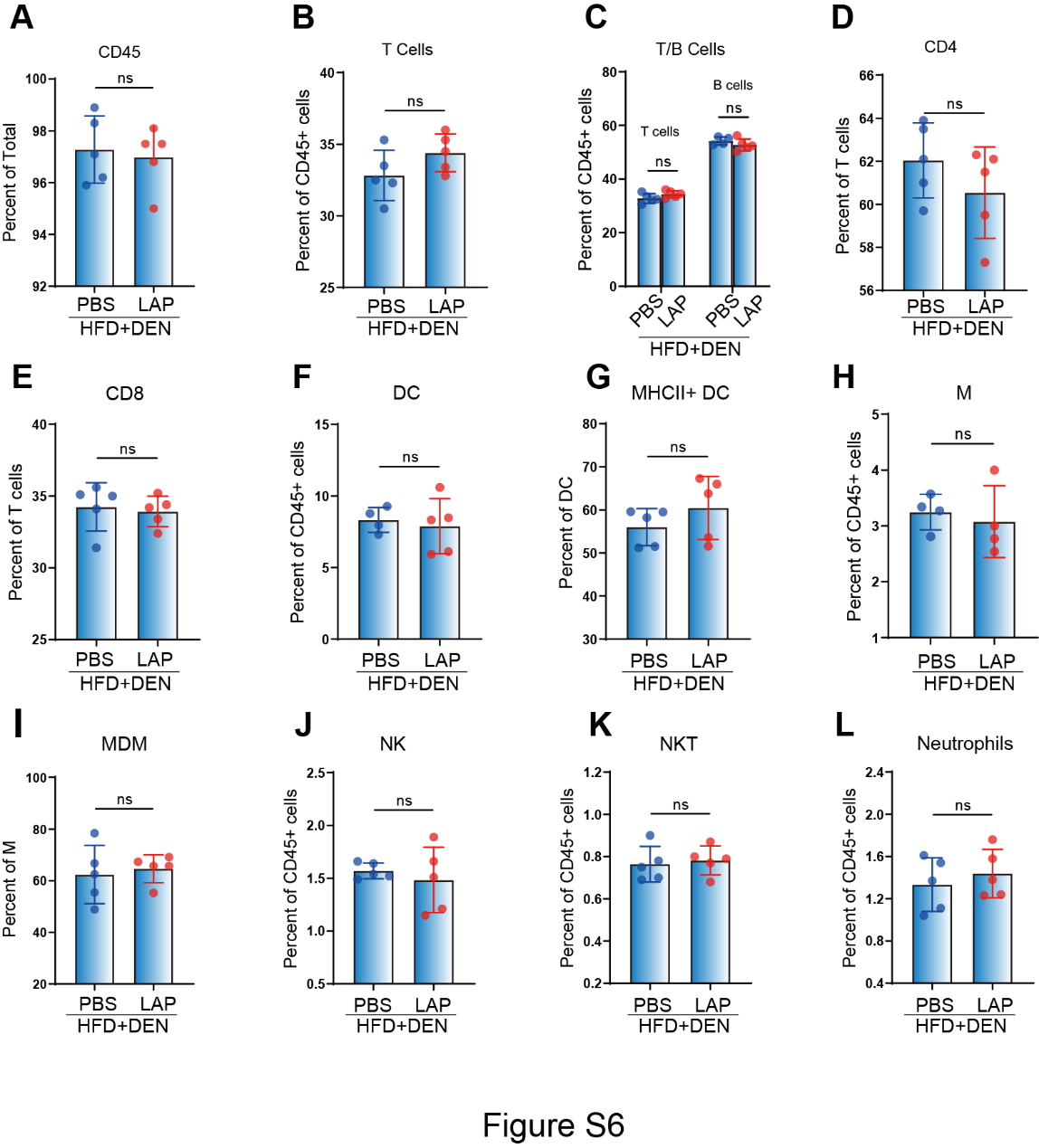


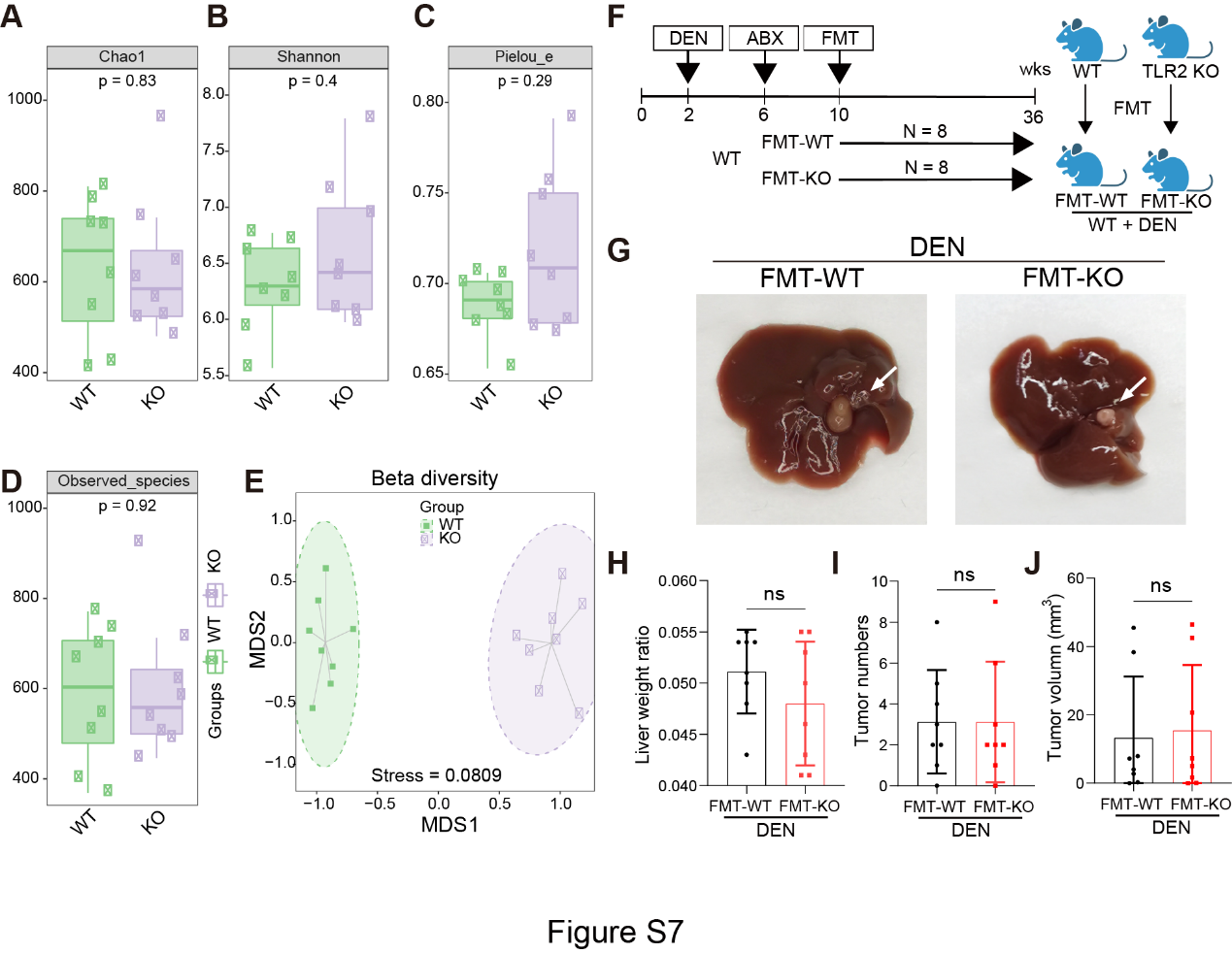


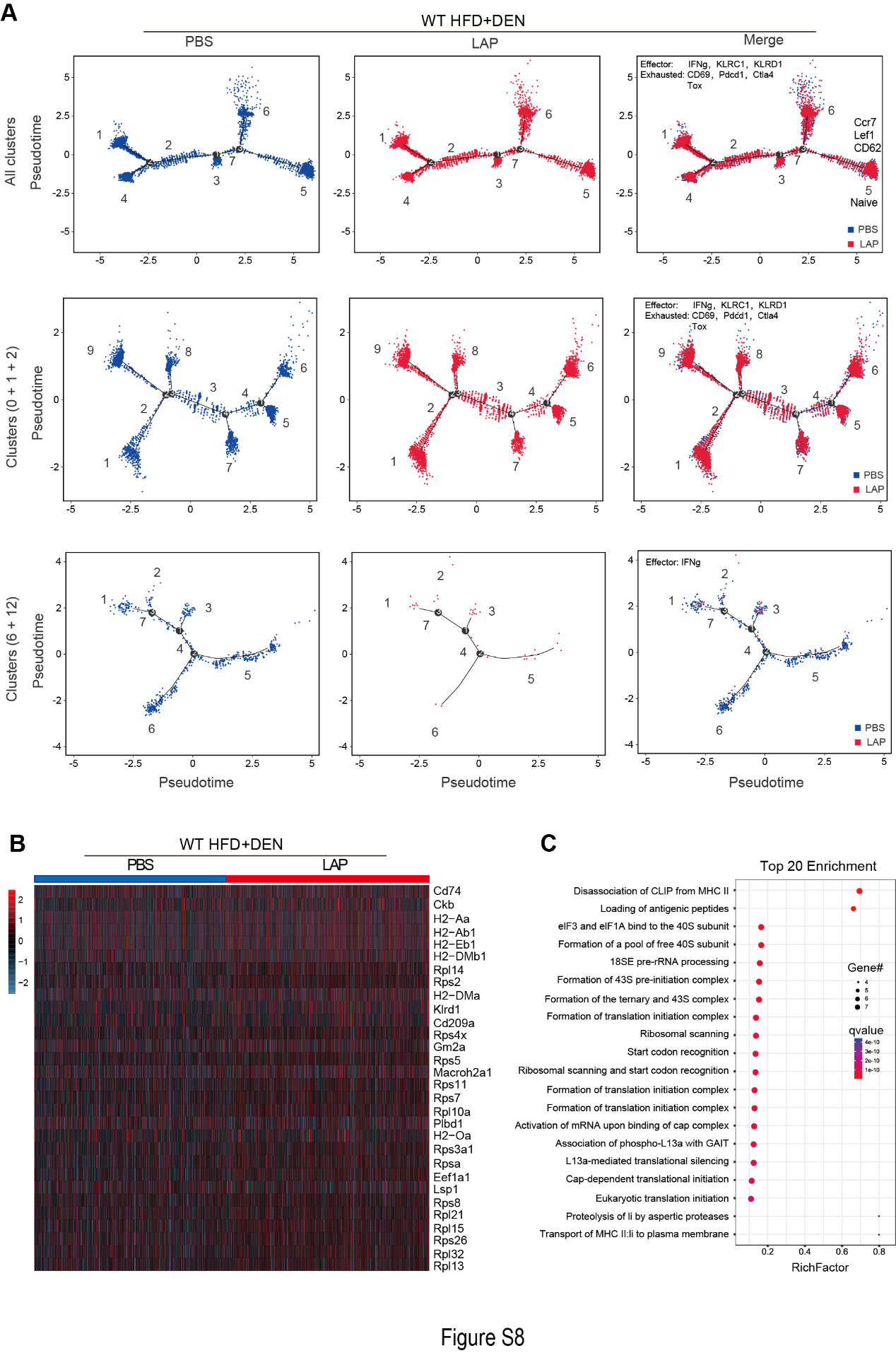

Supplement: Supplementary file 1 [file DataSheet1.docx]
